# Intravenous loss of over‐the‐wire catheter guidewires in 13 horses

**DOI:** 10.1111/jvim.16960

**Published:** 2023-12-14

**Authors:** Kallie J. Hobbs, Kimberly A. S. Young, Sara Nannarone, Daniela Luethy, Charlotte Hopster‐Iversen, Harold C. McKenzie, Elsa K. Ludwig

**Affiliations:** ^1^ Department of Large Animal Clinical Sciences North Carolina State University College of Veterinary Medicine Raleigh North Carolina USA; ^2^ Department of Veterinary Medicine University of Perugia Perugia Italy; ^3^ Department of Large Animal Clinical Sciences University of Florida College of Veterinary Medicine Gainesville Florida USA; ^4^ Department of Veterinary Clinical Sciences, Faculty of Health and Medical Sciences University of Copenhagen Taastrup Denmark; ^5^ Department of Large Animal Clinical Sciences Virginia Maryland College of Veterinary Medicine Blacksburg Virginia USA

**Keywords:** catheter, equine, guidewire, intravenous

## Abstract

**Background:**

Over‐the‐wire (OTW) catheter placement is performed frequently in horses. Intravascular loss of a guidewire has been anecdotally reported, but there is limited information regarding the treatment and outcome of horses that have experienced this complication of OTW catheter placement.

**Objectives:**

Describe the clinical and diagnostic features, treatment, and outcome of horses experiencing IV guidewire loss at the time of OTW catheter placement.

**Animals:**

Thirteen horses.

**Methods:**

Multicenter retrospective study to identify horses with IV guidewire loss. Horses of all ages were considered for inclusion. Horses were excluded from the study if complete medical records of signalment, indication, and outcome were not available. Intravenous guidewire loss was defined as the guidewire being lost IV at the time of OTW catheter placement.

**Results:**

No horses in this study experienced adverse clinical signs associated with the loss of a guidewire. Eight horses had the guidewire removed and the guidewire was left in situ in 5 horses. None of the horses with the guidewire in situ had experienced long‐term effects.

**Conclusions and Clinical Importance:**

Intravenous guidewire loss seems to have a good long‐term prognosis even in horses in which removal of the guidewire was not possible. Thus, in horses where guidewire removal is not feasible, guidewires that remain in situ may have limited to no adverse effects.

**Abbreviation**
OTW: over‐the‐wire

## 1 INTRODUCTION

Placement of an over‐the‐wire (OTW) catheter in horses is a commonly performed and relatively safe procedure, but it has been associated with several complications. One complication that can occur during OTW catheter placement is the IV loss of the catheter guidewire. Limited information is available regarding this OTW catheter placement complication, with only a single published case report on the IV loss of a guidewire and its subsequent removal in a horse.^1^ Anecdotal evidence suggests that IV guidewire loss may be a more common occurrence than is reported in the veterinary literature. This paucity of literature offers veterinarians little guidance regarding management and prognosis for horses experiencing loss of IV guidewires.

Many case reports in human patients have documented IV loss of a guidewire that occurred during OTW catheter placement or as an incidental finding at the time of other procedures.^2^, ^3^, ^4^, ^5^, ^6^ Commonly reported complications resulting from IV guidewire loss include cardiac dysrhythmias, thrombosis, embolism, guidewire perforation of vessels or heart chambers, infection, and fever of unknown origin.^3^, ^5^, ^6^, ^7^ The small size of the guidewire and straightforward surgical approach have made removal of a lost IV guidewire the standard of care in humans.^3^, ^8^

The aim of our retrospective study was to describe the clinical findings, progression, and outcome of IV loss of a guidewire in hospitalized horses. We have included cases in which the guidewire was left in situ, cases where the guidewire was removed, and recommendations for the management of IV guidewire loss.

## 2 MATERIALS AND METHODS

### 2.1 Case selection criteria

Intravascular loss of a guidewire was defined as any case in which the guidewire was inadvertently dropped IV at the time of OTW catheter placement. Horses of all ages were eligible for inclusion as long as detailed medical records (signalment, diagnosis, and case progression) were available.

### 2.2 Medical records review

Medical records from North Carolina State University were searched systematically for cases with reference to IV guidewire loss. Cases were identified by the search terms “guidewire” or “OTW catheter” and data were extracted from the records of cases meeting the inclusion criteria. Additional cases were identified through the American College of Veterinary Internal Medicine listserv, American College of Veterinary Surgeons listserv, and veterinary social media, with all respondents having the option to remain anonymous. Data obtained from medical records included signalment, presenting complaint, catheter size and placement location, clinical signs after the incident, advanced imaging results, whether or not guidewire retrieval was attempted and the outcome of retrieval, and case survival (Table S1).

### 2.3 Data analysis

Descriptive data were generated and results were reported as case number and percentage of cases.

## 3 RESULTS

### 3.1 Animals

Cases of 15 horses that had a guidewire lost IV into the right or left jugular vein during catheter placement were reviewed. Of the 15 cases, 13 horses met the inclusion criteria (Table S1). Two submitted neonatal foal cases were excluded from the study because of lack of case detail. The ages of the remaining 13 horses included in the study ranged from 1 to 19 years.

### 3.2 Catheter characteristics

All catheters of the included cases were 14‐gauge OTW catheters. Four of the catheters were placed in the right jugular vein (31%) and 9 were placed in the left jugular vein (69%). All catheters were placed in the direction of the heart.

### 3.3 Clinical abnormalities, diagnostic imaging, and guidewire localization

None of the horses had any abnormal clinical signs directly associated with OTW guidewire loss at the time of IV loss of the guidewire. Diagnostic imaging was performed in all cases and included radiography (n = 11), ultrasonography (n = 7), fluoroscopy (n = 1), and echocardiography (n = 7). Plain radiographs were used to successfully visualize the guidewire in 10 horses and the guidewires were located within the jugular vein in the region of the jugular groove in 7 of the 10 horses. Ultrasonography successfully identified guidewires within the jugular vein at the level of the thoracic inlet in 2 horses and within the jugular vein in the region of the jugular groove in 2 horses. Radiography and fluoroscopy were performed in 2 horses for guidewire localization. Seven horses underwent echocardiography, which identified no cardiac abnormalities. In 4 of the 7 horses that underwent echocardiography, the guidewire was located within the heart. In horse 1, the guidewire initially was located in the right atrium, but on repeat echocardiography 1 day later, the wire was found in left branch of the pulmonary artery in the caudal lung lobe. In horse 2, the guidewire was located in the right atrium and right ventricle. In horse 3, the guidewire traversed the tricuspid valve with the cardiac end lodged at the entrance to the right ventricle and based on ultrasonography the noncardiac end was located at the entrance of the cranial vena cava. In horse 4, the cardiac end of 1 guidewire was lodged in the apex of the right ventricle. Repeat echocardiography of 3 horses in which the guidewire remained in the heart indicated no cardiac changes or changes in guidewire location (horses 1, 2, and 3). Furthermore, several months after intravascular guidewire loss, exercising ECG was performed on 2 of the horses with intracardiac guidewires, with no arrhythmias found (horses 1 and 2).

### 3.4 Cases with successful guidewire retrieval

Guidewire retrieval was attempted in 12 of the 13 horses included in the study. Guidewire retrieval was successful in 8 cases (67% of horses that underwent attempted retrieval). Retrieval techniques included a percutaneous endovascular approach (n = 2) or venotomy (n = 6) and were performed either standing or under general anesthesia.

#### 3.4.1 Percutaneous endovascular approach

Two horses underwent a sedated, standing, percutaneous endovascular technique for guidewire retrieval (horses 7 and 8).^1^ The horses had 3 electrodes placed in base‐apex configuration to provide a continuous ECG and heart rate monitoring during the retrieval procedure. A 105 mm, 13‐gauge polypropylene IV catheter (Logicath Deltec, Smiths Medical, London, England) was placed in the affected jugular vein to facilitate guidewire removal. This catheter was placed under fluoroscopic guidance in 1 horse and under radiographic and ultrasonographic guidance in the second horse. In both horses, the guidewire was identified in the distal third of the jugular vein and a 4‐pronged retrieval instrument (Mercury Produzione S.r.l., Perugia, Italy) was introduced through the IV catheter and directed toward the lost guidewire. Once near the proximal end of the guidewire, the prongs of the retrieval instrument were opened and closed in order to grasp the guidewire. After several attempts, the guidewire was grasped approximately 2.5 cm from the proximal end and withdrawn through the jugular vein and to the distal end of the IV catheter. The IV catheter then was removed from the vein and both the guidewire and retrieval instrument were withdrawn from the vein at the site of the catheter insertion through a 3 cm skin incision. Manual compression was applied to the incision location for 10 minutes to achieve hemostasis.^1^

#### 3.4.2 Venotomy

Successful guidewire removal was performed in 6 horses via venotomy. Four horses (3 hospitalized horses and 1 horse in the field) underwent guidewire localization with diagnostic imaging, followed by guidewire retrieval via standing venotomy. Surgical venotomy technique was described in only 1 case, in which the guidewire was successfully removed under general anesthesia. This horse was anesthetized for exploratory celiotomy (presenting complaint of colic) and during surgery a magnet was used to stabilize the guidewire in the jugular vein. A small skin and venous incision (venotomy) were made over the stabilized guidewire, and the guidewire was grasped and removed percutaneously. The jugular vein and skin were sutured and the horse recovered uneventfully. A second horse underwent general anesthesia and guidewire retrieval was unsuccessfully attempted. After anesthesia recovery, the guidewire was removed the next day via standing venotomy. Only 1 horse suffered from postvenotomy complications, which included left facial swelling that resolved within several weeks and persistent left jugular vein thrombosis.

### 3.5 Cases with guidewires left in situ

Guidewire retrieval was unsuccessful in 5 of the horses included in this study, resulting in the guidewire remaining in situ.

#### 3.5.1 Horse 1

**FIGURE 1:**
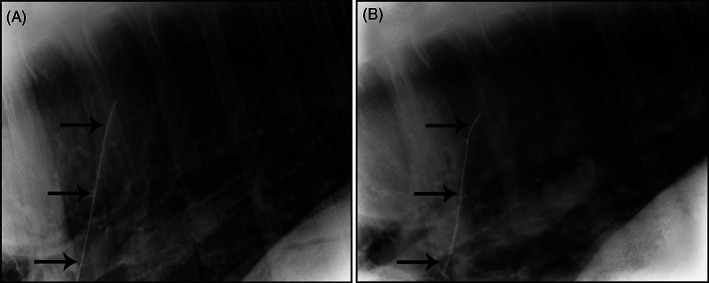
Thoracic radiographs of a 6‐year‐old Standardbred mare. The guidewire (black arrows) was located within the left pulmonary artery. (A) 24 hours after intravascular loss and (B) 2 years after intravascular loss.

Using echocardiography, the guidewire initially was visualized in the right atrium of the heart. With the horse sedated and standing, percutaneous endovascular guidewire retrieval was unsuccessfully attempted using biopsy forceps via the jugular vein. Throughout the procedure the horse remained clinically stable, but isolated atrial premature beats were observed on ECG. After unsuccessful retrieval, the guidewire was noted to have migrated to the left branch of the pulmonary artery of the caudal lung lobe (Figure 1A). A human medical cardiologist was consulted for advice, and it was decided that no additional invasive attempts were warranted. Three years later, the horse had no reported complications. Repeat echocardiography disclosed no cardiac abnormalities and the guidewire remained in the left branch of the pulmonary artery (Figure 1B). Because the horse was part of a research herd, performance during exercise was not evaluated.

#### 3.5.2 Horse 2

Thoracic radiographs and echocardiography located the guidewire coursing from the cranial vena cava into the right atrium, through the tricuspid valve, and into the right ventricle, with the J‐end of the guidewire situated in the right ventricular apex. Four days after the IV loss of the guidewire, retrieval was attempted under general anesthesia using radiographic and echocardiographic guidance. A 10‐French introducer catheter was inserted into the left jugular vein and removal of the guidewire was attempted using flexible tripod grasping forceps and a basket retrieval device. Removal was unsuccessful because of the position of the guidewire against the wall of the vena cava with the freely movable J‐end within the right ventricle. During hospitalization, serial echocardiograms indicated no changes to the cardiac architecture or guidewire location, with the J‐end remaining in the right ventricular apex. Three months after hospital discharge, reevaluation indicated no changes on echocardiography or thoracic radiographs. Nineteen months postincident, ECG indicated no pathologic cardiac arrhythmias at exercise (walk, trot, canter) and the horse remained at the previous level of performance with no reported complications.

#### 3.5.3 Horse 3

**FIGURE 2:**
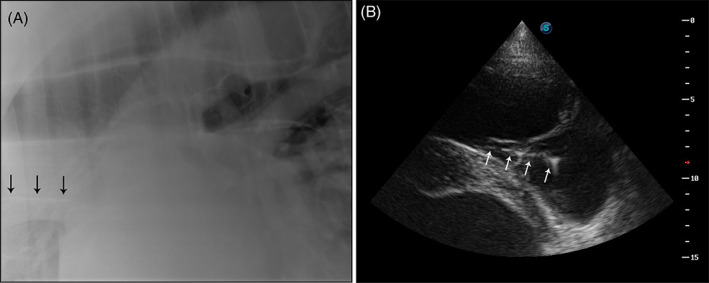
Diagnostic images localizing an intravascular guidewire in a 19‐year‐old Morgan mare. (A) thoracic radiograph with an increased opacity superimposed with the cranial vena cava (black arrows), likely the lost guidewire. (B) echocardiograph with guidewire traversing the tricuspid valve and to the ventricular inlet septum of the right ventricle of the heart (white arrows).

Immediately after IV loss, the guidewire could not be identified by peripheral venous ultrasonography. The next morning, the guidewire was ultrasonographically located in the right atrium of the heart, traversing the tricuspid valve. No structural heart changes were identified. A percutaneous endovascular retrieval under standing sedation using an introducer catheter placed in the right jugular vein was unsuccessful. During the procedure, the ECG was normal except for intermittent ventricular premature complexes that occurred when the guidewire was touched by the retrieval instrument. Four days later, a second unsuccessful retrieval was attempted. Echocardiography performed during the second retrieval attempt located the guidewire traversing the tricuspid valve, with the end lodged in the ventricular entrance (Figure 2). On thoracic radiographs, the guidewire was visualized in the cranial vena cava (Figure 2). Cardiac structure and ECG were unchanged from the previous retrieval attempt. The mare was maintained on PO clopidogrel (4 mg/kg loading dose followed by a 2 mg/kg maintenance dose, q24h) until reevaluation 1 month after the incident. At reevaluation, ECG was performed using a Holter monitor, and identified no ECG abnormalities at rest and the PO clopidogrel was discontinued. The mare returned to work (dressage) and was reported to perform normally. Approximately 3 years after the IV guidewire loss, the mare was euthanized because of colic. No necropsy was performed.

#### 3.5.4 Horse 4

Intravascular guidewire loss occurred in a pregnant mare hospitalized for an indolent ocular ulcer. Using echocardiography, the guidewire was found to be lodged in the apex of the right ventricle and a standing, sedated guidewire retrieval was unsuccessfully attempted using interventional radiology. The mare uneventfully delivered a normal foal, and no complications were reported during the mare's 6‐month period of hospitalization for the ocular condition. Five years after the incident, the mare was euthanized for reasons unrelated to the indwelling guidewire. No necropsy was performed.

#### 3.5.5 Horse 5

**FIGURE 3:**
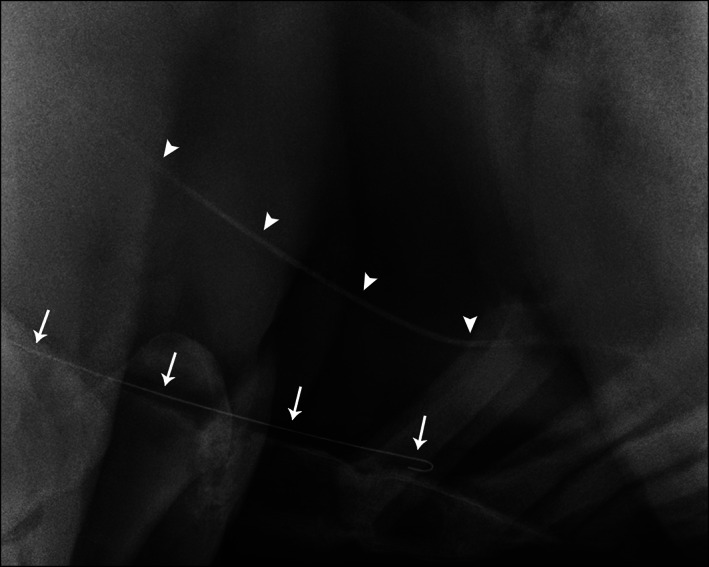
Thoracic radiograph of a 14‐year‐old Thoroughbred mare with guidewire located dorsal to the internal surface of the sternum, within the internal thoracic vein (white arrows). An OTW catheter placed within the lateral thoracic vein is visible dorsal to the guidewire (white arrowheads).

The guidewire was lost IV during OTW catheter placement that was performed in the field. On referral to the teaching hospital, the vital signs of the mare were all within normal limits. Radiography located the guidewire in the internal thoracic vein, rather than within the heart (Figure 3). Because the guidewire was deemed to be in a safe location, no retrieval was attempted, leaving the guidewire in situ. Eight months later, the mare was doing well with no reported complications.

### 3.6 Long‐term case outcomes

All horses included in the study survived to discharge. Guidewires remaining in situ did not appear to affect the ability of horses to return to their previous level of work (n = 4) or undergo successful parturition (n = 1). To our knowledge, only 1 horse with a retained guidewire removed suffered from a related complication, which was persistent left jugular vein thrombosis. At the time of writing, no other horses that underwent successful guidewire removal had any complications or long‐term adverse effects, but most horses were not followed after discharge from the hospital. The longest reported time interval from the time of incident to last follow‐up was 10 years (Table S1).

## 4 DISCUSSION

Eight of the 13 horses (62%) in our study underwent successful guidewire retrieval via either venotomy or fluoroscopy‐ or radiography‐guided percutaneous removal. In these 8 horses, the guidewires were lodged in the jugular vein, suggesting that there is a greater chance of retrieving guidewires located within the jugular veins than those located in the heart or surrounding vasculature. Three of these 8 horses underwent successful guidewire retrieval up to 4 days after IV loss. In humans, IV guidewires frequently are discovered years after loss during unrelated procedures, and with no reported clinical abnormalities or complications related to the in situ guidewire.^2^, ^3^, ^4^, ^5^, ^6^ In our study, all 5 horses with the guidewire left in situ were able to return to their previous level of activity with no complications. These findings suggest that IV guidewire loss in horses may not be as life‐threatening as anecdotally described.

In human medicine, OTW catheter placement is referred to as the Seldinger technique, and involves inserting an introducer needle into the vein, passing a guidewire through the needle to establish direct venous access, removing the needle while leaving the guidewire in place, passing the catheter over the guidewire and into the vein, and finally, removing the guidewire.^1^, ^4^, ^6^, ^9^ Although this catheterization technique is commonly performed and decreases the risk of air embolism and venous injury, it carries a complication rate of 12‐15%, including IV loss of guidewires.^3^, ^5^, ^6^, ^7^, ^10^ In most cases in humans, IV guidewire retrieval is achieved using interventional radiology techniques to grasp the guidewire using endovascular snares, forceps, or baskets.^3^, ^6^, ^10^ Occasionally, surgical venotomy may be required to facilitate retrieval.^6^, ^10^ However, in a small number of case reports in humans, guidewires have been left in situ when the risks to the patient associated with removal were deemed too high.^1^, ^2^, ^11^, ^12^

Similar to the situation in humans, IV guidewire loss in horses is a rarely reported complication of OTW catheter placement. Radiography was the most successful imaging modality for visualization of the lost guidewire (11 of the 11 cases where radiography was performed, 100%), whereas the guidewire was ultrasonographically identified in 4 out of the 7 cases (57%). These findings suggest that plain radiographs are a reasonable choice for a first diagnostic step in localization of the guidewire. In humans, radiographs are the standard of care for localization of the guidewire after IV loss.^7^, ^12^, ^13^

Limitations of our retrospective study include the lack of long‐term follow‐up in several of the horses. Because these horses underwent successful removal of the guidewire, the lack of follow‐up information decreases our understanding of the long‐term prognosis for horses recovering from IV guidewire retrieval. Additionally, because of the retrospective nature of the study and the use of listservs to anonymously acquire cases, several case records lacked sufficient details, preventing statistical comparison of the different treatment techniques and associated case outcomes.

Our findings suggest that although the IV loss of a guidewire is a serious complication, the prognosis for survival is excellent, and all horses in our study survived to hospital discharge. Immediate guidewire retrieval may not be necessary, because 3 horses underwent successful guidewire removal several days after IV loss. In these 3 horses, the guidewire did not migrate after becoming lodged at the initial resting point, and these horses did not have any complications associated with the guidewire. This observation may indicate that once the guidewire is lodged, it is unlikely to move, and therefore planning for the appropriate surgical approach may be more important than immediate guidewire removal. Additional findings of our study suggest that plain radiography may be the best diagnostic imaging modality for identification of the lost IV guidewire, because guidewires were located on 100% of plain radiographs when radiography was performed. Other types of diagnostic imaging proved to be less effective for identifying lost guidewires or required specialized equipment and training to perform. Finally, of the 8 cases with successful guidewire removal, only 1 horse suffered from a persistent complication postretrieval (jugular vein thrombosis). None of the horses with the guidewire left in situ suffered any associated complications. Overall, IV guidewire loss during OTW catheter placement in horses might be a less severe complication than anecdotally described.

## CONFLICT OF INTEREST DECLARATION

Authors declare no conflict of interest.

## OFF‐LABEL ANTIMICROBIAL DECLARATION

Authors declare no off‐label use of antimicrobials.

## INSTITUTIONAL ANIMAL CARE AND USE COMMITTEE (IACUC) OR OTHER APPROVAL DECLARATION

Authors declare no IACUC or other approval was needed.

## HUMAN ETHICS APPROVAL DECLARATION

Authors declare human ethics approval was not needed for this study.

## Supporting information


**Table S1.** Summary of patient signalment, presenting complaint, affected jugular vein, diagnostic imaging, guidewire location, guidewire retrieval, complications, and survival for the 13 horses included in this retrospective study. No significant findings are reported as NSF.
