# Supplementary material for: Intravenous loss of over‐the‐wire catheter guidewires in 13 horses
Source: J Vet Intern Med. 2023 Dec 14;38(1):411–6. doi: 10.1111/jvim.16960 (PMC10800200; doi:10.1111/jvim.16960)
Supplement: Supplementary file 1 — Table S1. Summary of patient signalment, presenting complaint, affected jugular vein, diagnostic imaging, guidewire location, guidewire retrieval, complications, and survival for the 13 horses included in this retrospective study. No significant findings are reported as NSF. [file JVIM-38-411-s001.pdf]

Horse Number	Age (years)	Breed	Gender	Presenting Complaint	OTW Catheter Size	Jugular Vein Affected (R vs. L)	Diagnostic Imaging Performed (Y/N)				Guidewire Location	Retrieval Attempted? (Y/N)	Retrieval Approach (Y/N) & Success?		Wire in situ? (Y/N)	Recheck Examinations and Complications?	Survival to Hospital Discharge (Y/N)	Long-Term Survival
							Radiography	Ultrasonography	Fluoroscopy	Echocardiography: Number Performed and Findings			Percutaneous Endovascular	Venotomy				
1	6 yo	Standardbred	Mare	Research	14 g	R	Y: thoracic	N	N	Y: 1) guidewire originally located in right atrium, 2) guidewire migrated to caudal lung lobe's left branch of the pulmonary artery	Caudal lung lobe's left branch of the pulmonary artery	Y: standing, sedated	Y: not successful	N	Y	Recheck echocardiography revealed no obvious cardiac abnormalities and guidewire remained in caudal lung lobe's left branch of the pulmonary artery. No abnormalities noted on working echocardiogram, no reported complications	Y	Currently alive three years post incident
2	12 yo	Quarter Horse	Gelding	Botulism	14 g	L	Y: thoracic	Y	N	Y: 1) NSF, 2) NSF	Coursing from cranial vena cava into right atrium, through tricuspid valve, and into right ventricle. J-end of guidewire situated in right ventricular apex	Y: general anesthesia	Y: not successful	N	Y	Serial echocardiograms revealed no changes to cardiac architecture or guidewire location. 3 months post discharge from hospital: no changes on echocardiography or thoracic radiographs. No pathologic cardiac arrhythmias noted during working ECG	Y	Currently alive and performing at previous level of work 19 months post-incident
3	19 yo	Morgan	Mare	Colic	14 g	L	Y: thoracic	Y: located guidewire at level of thoracic inlet	N	Y: 1) guidewire identified in right atrium, 2) cardiac end of guidewire was traversing the tricuspid valve and lodged at entrance of right ventricle. Non-cardiac end guidewire located at entrance of cranial vena cava	Cardiac end of guidewire was traversing the tricuspid valve and lodged at entrance of right ventricle. Non-cardiac end guidewire located at entrance of cranial vena cava	Y: standing, sedated	Y: attempted twice, not successful	N	Y	None reported	Y	Survived three years following discharge from hospital, euthanized due to reasons unrelated to in situ guidewire
4	Adult (age not reported)	Warmblood	Mare	Indolent ocular ulcer	14 g	L	Y	Y	N	Y: 1) guidewire located in apex of right ventricle	Apex of right ventricle	Y: standing, sedated with interventional radiography	N	Y: not successful	Y	Owners reported increased lethargy at turnout, however horse was retired following the incident. All follow-up examinations with the attending veterinarian were normal and the mare successfully foaled post-incident	Y	Survived for five years post incident, euthanized due to causes unrelated to intravascular guidewire
5	14 yo	Thoroughbred	Mare	Referred for intravenous loss of guidewire	14 g	L	Y: located guidewire in internal thoracic vein	N	N	N	Internal thoracic vein	N	n/a	n/a	Y	None reported	Y	Currently alive at time of publication
6	14 yo	Thoroughbred	n/a	Research	14 g	L	Y	N	N	Y: 1) NSF	Left jugular vein	Y: standing, sedated; in field	N	Y: successful	N	None reported	n/a: horse not referred to hospital.	Survived for 10 years post incident, died due to causes unrelated to intravascular guidewire
7	17 yo	Arabian	Stallion	Urethral Bleeding	14 g	L	Y	Y	NO	Y: 1) NSF	Affected jugular vein	Y: standing, sedated	Y: successful	N	N	None reported	Y	Currently alive two years post incident
8	17 yo	Dutch Warmblood	Mare	Dystocia	14 g	R	Y	Y	Y	N	Distal third of the affected jugular vein	Y: standing, sedated	Y: successful	N	N	None reported	Y	Horse was alive at 6 months post discharge from hospital, ultimately lost to long-term follow up
9	3 yo	Quarter Horse	Mare	Colic	14 g	L	N	Y: used during venotomy	N	N	Left jugular vein	Y: standing, sedated	N	Y: successful	N	None reported	Y	Survived three years following discharge from hospital, then lost to long-term follow up
10	11 yo	Irish Hunter Sport Horse	Gelding	Colic	14 g	R	N	Y: used during venotomy	N	N	Right jugular vein	Y: standing, sedated	N	Y: successful	N	None reported	Y	Horse returned to original level of work six months after discharge from hospital. Lost to long-term follow up
11	1 yo	Warmblood	Mare	Colic	14 g	L	Y	N	N	N	Left jugular vein	Y: general anesthesia during colic surgery	N	Y: successful	N	None reported	Y	Survived 10 years following incident, lost to long-term follow up
12	14 yo	Warmblood	Gelding	n/a	14 g	R	Y	N	N	N	Right jugular vein	Y: standing, sedated	N	Y: successful	N	None reported	Y	Lost to long-term follow up
13	16 yo	Pony	Gelding	Sinus mass	14 g	L	Y: located guidewire at level of thoracic inlet	Y: located guidewire at level of thoracic inlet	N	Y: 1) NSF	Left jugular vein, level of thoracic inlet	Y: first attempt general anesthesia; second attempt standing, sedated	N	Y: first attempt unsuccessful, second attempt successful	N	Left jugular vein thrombosis	Y	Survived 7 months following discharge from hospital, lost to long-term follow up

Supplementary Table 1. Summary of patient signalment, presenting complaint, affected jugular vein, diagnostic imaging, guidewire location, guidewire retrieval, complications, and survival for the 13 horses included in this retrospective study. No significant findings are reported as NSF.
